# Telephone follow-up as a substitute for standard out-clinic follow-up in CPAP therapy for obstructive sleep apnea patients: a randomized controlled trial

**DOI:** 10.1007/s11325-024-03045-w

**Published:** 2024-05-08

**Authors:** Sofie Krogh Wolsing, Jannie Christina Frølund, Christine Dalgård, Ole Hilberg, Eline Gantzhorn

**Affiliations:** 1 Department of Medicine, Lillebaelt Hospital, Vejle Hospital, University Hospital of Southern Denmark, Beriderbakken 4, 7100 Vejle, Denmark; 2 Department of Regional Health Research, University of Southern Denmark, Odense, Denmark; 3 Research Unit of Clinical Pharmacology, Pharmacy and Environmental Medicine, University of Southern Denmark, Odense, Denmark

**Keywords:** Obstructive sleep apnea, Continuous positive airway pressure, Telemedicine, Telephone consultation, Out-clinic

## Abstract

**Purpose:**

This study assessed the feasibility of telephone follow-up consultations (TC) using an online data sharing and editing function (Airview™), as alternative to standard out-clinic follow-up consultations (SC) on adherence to continuous positive airway pressure (CPAP) in obstructive sleep apnea (OSA) patients. Furthermore, we investigated compliance to follow-up consultations and examined potential influencing factors, including baseline AHI (apnea–hypopnea-index), age, and distance from home to the hospital on consultation compliance.

**Methods:**

Two hundred OSA patients, with AHI $\ge$ 5 were randomly assigned (1:1) to receive TC or SC with follow-up after one month and 12 month of CPAP initiation. Adherence goal was defined as achieving $\ge$ 4 h of CPAP use daily in 70% of the days in a 365-days period.

**Results:**

The proportion of participants achieving CPAP adherence was non-significantly lower in the TC group compared to the SC group (TC: 30% versus SC: 36%, adjusted OR 0.84, *p* = 0.59). Of participants who completed the study, the TC group had a significant average of 107 min less use of CPAP compared to the SC group (*p* = 0.048). However, a higher proportion of participants was compliant to consultations in the TC group. The only influencing factor found was increasing baseline AHI, which might be a predictor for compliance to consultations and adherence to CPAP therapy.

**Conclusion:**

TC might serve as substitute for SC in some part of the OSA population. If TC becomes a part of CPAP therapy management, it is important to consider patient characteristics and treatment-related issues to prevent decline in adherence.

**Supplementary Information:**

The online version contains supplementary material available at 10.1007/s11325-024-03045-w.

## Introduction

Obstructive sleep apnea (OSA) is a common and underdiagnosed sleep disorder, characterized by intermitted breathing pauses during sleep due to partially or complete collapse of the upper airway tissue [1–3]. This condition results in a fragmented sleep pattern, exerting a profound impact on the physical and mental health of the individual with OSA. OSA is associated with the risk of cardiovascular disease, stroke, type 2 diabetes, depression, cognitive impairment, and the risk of car accidents during driving. Left untreated, OSA escalates the risk of mortality [1, 2]. Risk factors contributing to the development of OSA are male sex, increasing age, obesity, craniofacial differences, ethnicity, and genetic predisposition [3, 4]. The estimated prevalence of symptomatic OSA consisting of daytime sleepiness and snoring are 4% for men and 2% for women in Denmark [5]. The diagnostic criterion for OSA is the apnea–hypopnea-index (AHI), which measures the average apnea and hypopnea periods per hour of sleep. OSA is classified in mild (5–14 AHI), moderate (15–29 AHI) and severe (≥ 30 AHI) categories [6].

Continuous positive airway pressure (CPAP) therapy during sleep is the gold standard treatment for OSA [7]. The essence of CPAP therapy comprises delivering a consistent airflow through a mask, ensuring the upper airway remains unobstructed [8]. However, the efficacy of CPAP therapy materializes only when individuals consistently use the device, emphasizing the necessity for long-term treatment commitment [7]. Despite the proven clinical benefits of CPAP in OSA treatment, a significant challenge arises in adherence. Research indicates that 30% to 86% of individuals with OSA underutilize or entirely disregard their CPAP therapy. This nonadherence is attributed to discomfort during device use or a perceived lack of effectiveness [9, 10]. In the Danish context, almost half of individuals initiating CPAP therapy discontinue within the first year [11]. Addressing these adherence barriers is crucial in ensuring its effectiveness in the long-term management of OSA.

The demographic challenges looming over Denmark forecast a surge in activity within specialty out-clinics. Though, resources are not proportionally increasing, leaving the hospitals under pressure [12]. In response to this imbalance, telemedicine emerges as a necessary solution for innovating the healthcare system [7, 9]. In recent years multiple international studies has investigated telemedicine´s potential to enhance CPAP adherence for individuals with OSA. These studies explore various interventions, from automated feedback messages and continuous telemonitoring to telephone consultations and online education programs [1, 7, 13–15]. The present study aims to:

- Assess the feasibility of Telephone Follow-Up Consultations (TC) as alternatives to Standard Out-Clinic Follow-Up Consultations (SC) without compromising CPAP Adherence.
- Investigate which intervention yields the highest compliance to follow-up consultations.
- Explore potential influencing factors: Examining the impact of baseline AHI, age, and distance from home to the hospital on consultation compliance.

## Methods

This prospective, assessor blinded, (1:1) randomized controlled trial was initiated and conducted at the pulmonary clinic, Vejle Hospital, Denmark.

### Participants

Eligible participants were newly diagnosed OSA patients (AHI ≥ 5 events/h) aged 18 and above, who started CPAP therapy at the pulmonary clinic. Patients were referred to the clinic either by their general practitioner or a private otolaryngologist, with diagnostics and treatment expenses covered by the state, ensuring no cost to the patient [16]. Participants were recruited during the diagnostic process in the period November 2018 to August 2019. All participants gave written informed consent.

Inclusion criteria was the diagnosis obstructive sleep apnea. Exclusion criteria were OSA patients with; chronic obstructive pulmonary disease, need of bilevel treatment, isolated severe upper airway resistance syndrome (UARS) but normal AHI (< 5), long term non-invasive ventilation treatment, and those unable to comprehend or communicate in Danish.

### Randomization

The randomization procedure was conducted by one specialist doctor during the initial enrollment consultation when CPAP therapy was initiated. The allocation followed a 1:1 ratio in block size of 50. This procedure was implemented to maintain the integrity of the randomization in the event of an early study termination. Each participant selected an identical, concealed, non-transparent envelope from a box, containing a written note indicating either ‘intervention’ or ‘control’. Subsequently, participants were randomly assigned to either the intervention group, receiving telephone follow-up consultations (TC), or the control group, undergoing standard out-clinic follow-up consultations (SC).

Only the assessor responsible for the final data management and analysis remained blinded to the allocation of the participants until the primary analysis had been conducted.

### Interventions

At the initiation of the study, all participants received the ResMed, Airsense S10, AutoSet CPAP device, a mask, and other relevant applications. A nurse from the pulmonary clinic provided comprehensive instructions and guidance on the CPAP device. The patient tried on the device to find the minimum pressure settings needed for comfort. However, as the CPAP device have automatically adjustable settings, there were no need for monitoring and adjusting the device in a sleep lab.

Follow-up consultations occurred after one month and approximately 12 months from the study entry date. Due to different clinical circumstances, including the COVID-19 pandemic, shortage of nursing staff and cancellations of follow-up consultations, some of the follow-ups were delayed.

In the TC group, participants underwent follow-up consultations conducted over the telephone. The consultation nurse accessed sleep data using the CPAP device’s Airview™ function, which transmits data to the clinic. If issues arose with the mask or device, the nurse could provide assistance remotely and adjust settings online.

In the SC group, participants underwent standard out-clinic follow-up consultations as scheduled. Participants were instructed to bring the CPAP device along with the memory card and other applications to the consultation at the hospital. Adjustments to CPAP settings and the mask could be made during theses in-person consultations.

Throughout the study, all participants had the option to request additional consultations, either by telephone or at the clinic, primarily based on their assigned group.

#### Data collection

At baseline, participants underwent a clinical examination, including weight, height, and AHI. Baseline AHI was determined through cardiorespiratory monitoring during a single night of sleep at home. Participants were required to complete a questionnaire focusing on participant characteristics and the validated Epworth Sleepiness Scale (ESS), which assesses daytime sleepiness through eight questions, assigning a score [17] (Table 1).

**Table 1:** Baseline demographics and sleep data for participants allocated to TC or SC, and for participants in the TC or SC group in the complete case analysis

	Allocated to treatment population		Complete case population	
	SC	TC	SC	TC
*n* (%)	100 (50.0)	100 (50.0)	64 (45.7)	76 (54.3)
Sex (female)	28 (28)	25 (25.0)	16 (25.0)	17 (22.4)
Age (years)	56.1 (12.4)	54.4 (12.2)	57.7 (12.3)	54.9 (11.8)
BMI (kg/m^2^)	31.7 (5.2)	31.6 (5.6)	32.3 (5.3)	31.6 (5.6)
Distance to hospital (km)	27 (20–38)	27 (14–36)	28 (23–43)	25 (13–36)
Missing	1 (0.5)	1 (0.5)	1 (1.6)	0 (0)
AHI (events/hours)	32.5 (19.5–43)	25 (15–44.5)	34.5 (29–48)	31 (19–51)
ESS score	9.0 (4–12)	9.0 (5–13)	9.0 (5.5–12)	9.0 (5–12)
Missing	0 (0)	1(1)	0 (0)	1 (1.3)

Continuous variables are mean (SD) or median (25th-75th percentiles), categorical variables and missing are *n* (%)

*Abbreviations*: *SC* standard out-clinic follow-up consultation, *TC* telephone follow-up consultation, *AHI* apnea–hypopnea-index, *ESS* Epworth sleepiness scale

During the follow-up consultations, sleep apnea data including the average percentage of days meeting the adherence goal ($\ge$ 4 h of CPAP), the average minutes of CPAP usage, and the AHI were obtained using the Airview™ function in the CPAP device in the TC group, or the data from the memory card in the CPAP device was uploaded manually in the SC group. Sleep data, ESS questionnaire responses, and the number of extra consultations were collected at both follow-up consultations.

### Outcomes

The primary outcome was adherence to CPAP therapy over a 12-month period. Adherence was measured using the standard clinical definition outlined by Medicare, which requires an average of at least 4 h of CPAP usage per night for a minimum of 70% of the time over a consecutive 30 days period within the initial 3 months of initiation [7]. Though, in this study, the calculation of 70% usage is based on all 365 days.

A secondary outcome was the median CPAP usage in both minutes and percentage. Other secondary outcomes involved assessing the compliance to consultations, specifically exploring which consultation method had the lowest dropout rate over the entire study duration. We also investigated whether baseline AHI, age, and distance from home to the hospital influenced compliance to consultations and to what extend each group received additional consultations.

### Sample size

We hypothesized that there would be no significant distinction in adherence to CPAP therapy between TC and SC. By referencing a prior study [18], which assumed an estimated effect size of $\ge$ 1 h and a standard deviation of 1.75, our replication, considering an α risk of 0.05 and β risk of 0.2 in a two-sided test, indicated a necessity for 96 participants. However, recognizing the substantial dropout rates observed clinically during the first year of CPAP therapy, we included 200 participants.

### Statistical methods

For the examination of adherence to CPAP therapy, we performed an intention-to-treat approach using a logistic regression model adjusted for sex, age, baseline ESS, BMI, baseline AHI, and distance from home to hospital. In this analysis, participants lost to follow-up and dropouts were considered as nonadherent to CPAP therapy.

For the analysis of CPAP usage, we conducted a complete case analysis using a quantile regression model, adjusting for sex, age, baseline ESS, BMI, baseline AHI, and distance from home to the hospital. The assessment of compliance to consultations was also conducted as complete case analysis. Both the compliance and dropout analyses utilized a quantile regression model, Pearsons chi-squared test for categorical variables, and Students *t* test for parametric data and Wilcoxon Ranksum test for non-parametric data in the case of continuous variables.

A *P*-value < 0.05 was considered significant. All statistical analyses were performed using Stata/BE version 17.0 software (StataCorp, College Station, TX, USA).

## Results

### Study population

**Fig. 1:**
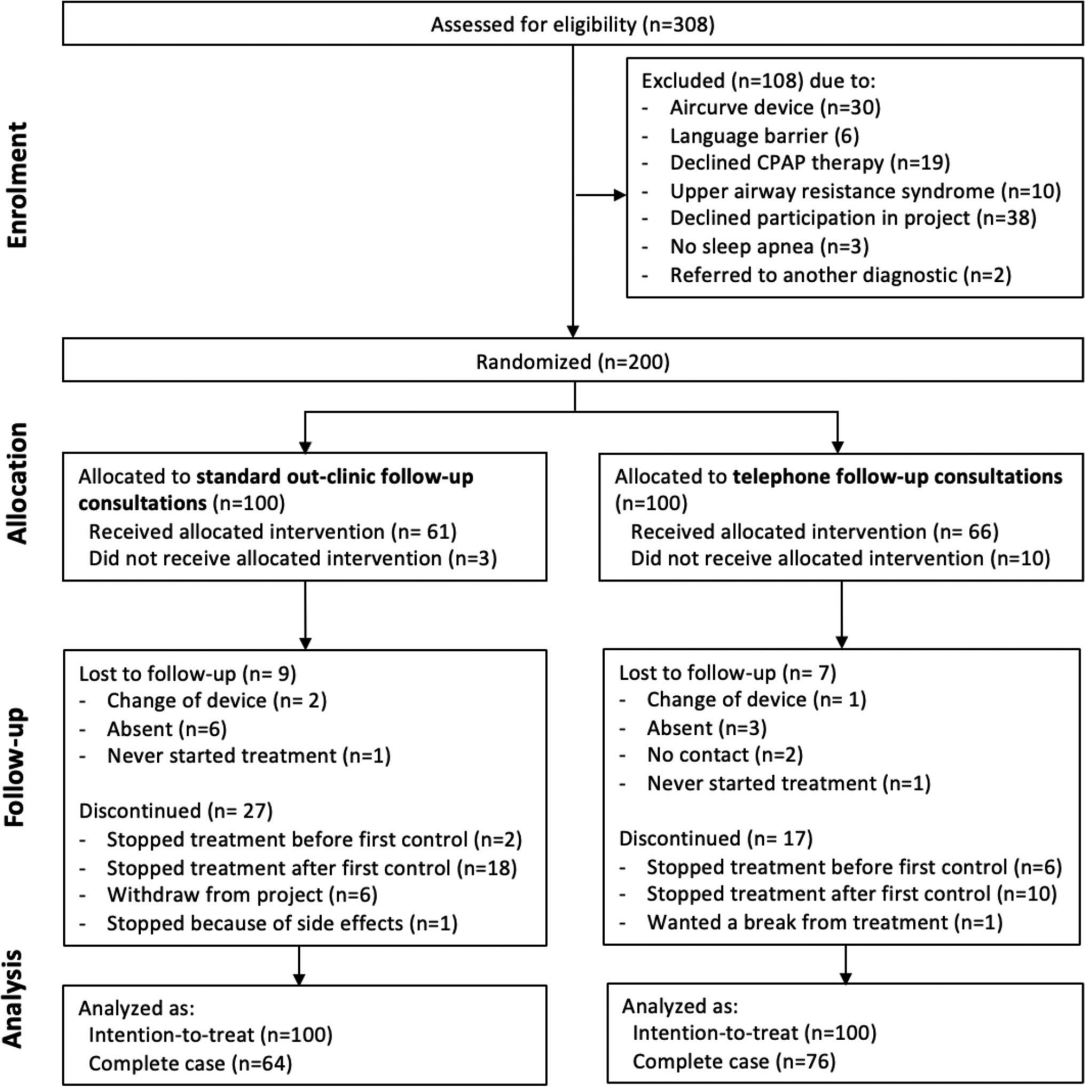
Flow chart illustrating the participant flow

Of the 308 patients assessed for eligibility between November 2018 to August 2019, 200 participants were randomized and received either TC (*n* = 100) or SC (*n* = 100). During the follow-up period, 60 participants dropped out of the study due to either loss to follow-up, or discontinuation of treatment or study participation (Fig. 1).

Table 1 presents baseline demographics and sleep data for all 200 participants allocated to either TC or SC, as well as for the participants in the complete case analysis (*n* = 140). Overall, the participants were mostly middle aged (55.2 years $\pm$ 12.3), male (73.5%), and overweight or obese (BMI 31.7 kg/m^2^ $\pm$ 5.4). The average distance from home to hospital was 30.5 km ($\pm$ 23.9), while the AHI indicated average severe OSA (33.8 AHI $\pm$ 20.9) and an average moderate ESS score (8.8 points $\pm$ 4.7).

### Assessing feasibility of TC as alternative to SC

When assessing adherence as the average percentage of days meeting the adherence goal ($\ge$ 4 h of CPAP in 70% of 365 days), the SC group demonstrated a 36% achievement rate, compared to 30% in the TC group. The crude OR was 0.76 (95% CI 0.42–1.38, *p* = 0.38) favoring the SC group. However, in the adjusted analysis, the OR changed to 0.84 (95% CI 0.45–1.58, *p* = 0.59). Both analyses indicated no significant difference between the TC and SC group (Table 2).

**Table 2:** Odds Ratio and 95% confidence interval for obtaining adherence goal^a^ over 12 months in the intention-to-treat analysis

	SC (*n* = 100)	TC (*n* = 100)	Crude OR (95% CI)	*P*-value	Adjusted^b^ OR (95% CI)	*P*-value
Adherence goal (%)^c^	36 (36)	30 (30)	0.76 (0.42–1.38)	0.38	0.84 (0.45–1.58)	0.59
Adherence goal (min)	47 (47)^d^	46 (46)	0.96 (0.55–1.67)	0.89	1.08 (0.59–1.96)	0.81

Numbers are *n* (%)

*Abbreviations*: *SC* standard out clinic follow-up consultation, *TC* telephone follow-up consultation, *CI* confidence interval

^a^Adherence goal measured as $\ge$ 4 h of CPAP use in 70% of 365 days

^b^Adjusted for sex, age, baseline ESS, BMI, baseline AHI, distance from home to hospital in kilometers

^c^Days of adherence goal obtained in percentage

^d^One value missing in the SC group for minutes (total *n* = 99)

The crude median difference in average daily CPAP use measured in percentage of the complete case population revealed a statistically significant difference of 25% (95% CI -49.42– -0.58, *p* = 0.045) in favor of the SC group. In the adjusted analysis, the median difference remained at 22% (*p* = 0.048). Additionally, in the adjusted analysis of minutes of CPAP use, the median difference was -107.7 min (95% CI -193.2– -22.1, *p* = 0.014), favoring the SC group (Table 3).

**Table 3:** Median difference and 95% confidence interval for CPAP use over 12 months in the complete case analysis

	SC (*n* = 64)^a^	TC (*n* = 76)	Crude median difference (95% CI)	*P*-value	Adjusted^b^ median difference (95% CI)	*P*-value
CPAP use (%)^c^	77.5 (29.5–93)	53 (11–87.4)	-25.0 (-49.4– -0.6)	0.045	-21.96 (-43.76– -0.15)	0.048
CPAP use (min)	324 (131–402)	235 (52–374.5)	-89.0 (-187.7–9.7)	0.077	-107.7 (-193.2– -22.1)	0.014

Numbers are median (25th-75th percentiles)

*Abbreviations*: *SC* standard out-clinic follow-up consultation, *TC* telephone follow-up consultation, *CI* confidence interval

^a^One value missing in the SC group for minutes (total *n* = 63)

^b^Adjusted for sex, age, baseline ESS, BMI, baseline AHI, distance from home to hospital in km

^c^Days of adherence goal obtained in percentage

### Assessment of compliance to follow-up consultations and potential influencing factors

The TC group had a higher compliance rate to the 12-months follow-up consultation, with 76% of participants completing the study, compared to 64% in the SC group. Participants in the TC group had a median of one extra telephone consultation between first month and 12-months follow-up, while the SC group had a median of 0.5 extra at-hospital consultations during the same period. Both differences were nonsignificant (Table 4).

**Table 4:** Median difference and 95% confidence interval for extra consultations between SC and TC groups in the complete case analysis

	SC	TC	Median difference (95% CI)	*P*-value
*n* (%)	64 (45.7)	76 (54.3)		
Extra consultations				
Number of extra phone consultations^a^	0 (0–1)	1(0–2)	1 (0.31–1.69)	0.005
Number of extra at-hospital consultations^a^	0.5 (0–1)	0 (0–1)	-1.00 (-1.45- -0.55)	0.09

Numbers are *n* (%) and median (25th-75th percentiles)

*Abbreviations*: *ESS* Epworth Sleepiness Scale, *AHI* apnea–hypopnea-index, *SC* standard out-clinic follow-up consultation, *TC* telephone follow-up consultation

^a^Number of extra consultations are measured between 1 month follow-up until 12 months follow-up

A dropout-analysis showed a non-significant median age difference of 2 years between participants who completed the study (56.5 (IQR 48–64.5)) and those who dropped out (IQR 54.5 (44–61.5)). The distance from home to the hospital did not differ significantly between participants who dropped out, and those who completed the study (Table 5). We performed an additional analysis which showed no difference in adherence to treatment between sex. Notably, the only significant difference between the groups was observed in baseline AHI (*p* < 0.001). Participants who completed the study had a median baseline AHI of 33 (IQR 21–49.5) compared to 18.5 (IQR 13.5–33.5) in the group who dropped out (Supplementary Document 1).

**Table 5:** Influence of age and distance to hospital on the dropout rate

	Complete case	Dropped out	Median difference (95% CI)	*P*-value
*n* (%)	140 (70.0)	60 (30.0)		
SC group	64 (64)	36 (36)		
TC group	76 (76)	24 (24)		
**Age**	56.5 (48–64.5)	54.5 (44–61.5)	-2.0 (-6.44–2.44)	0.38
SC group	59 (50–65.5)	54 (44–61.5)	-5.0 (-11.28–1.28)	0.12
TC group	54.5 (47–62)	55 (44–61.5)	0.0 (-7.19–7.19)	0.99
**Distance to hospital (km)**	27 (18–37)	27 (15–34)	0.0 (-5.38–5.38)	0.99
SC group	28 (23–43)	25.5 (14–33.5)	-3.00 (-10.58–4.58)	0.43
TC group	25 (13–36)	29 (15–38)	4.00 (-6.59–14.59)	0.46

Numbers are *n* (%) and median (25th-75th percentiles)

*Abbreviations*: *km* kilometers, *CI* confidence interval, *SC* standard out-clinic follow-up consultation, *TC* telephone follow-up consultation

## Discussion

In this randomized study, allocation to telephone follow-up consultations indicated a slight non-significant decrease in CPAP adherence compared with the standard out-clinic follow-up consultation. When measuring adherence as average minutes of CPAP use, no significant difference emerged between the groups.

While this study aligns with other studies demonstrating no minute-based differences [7, 13, 19], a meta-analysis suggested telemonitoring devices with active features could impact positively on adherence to CPAP therapy [20]. Another study only showed a significant increase in adherence within the first months, whereafter adherence became non-significant in the evaluation after 12 months [21]. Unlike the present study, previous studies incorporate more active telemonitoring devices, utilizing automated messages or continuous personnel contact to address non-adherence effectively [1, 7, 13, 14]. In contrast, our study relied solely on telephone consultations as substitute for in-person visits, supplemented by the Airview™ function for data collection during consultations. This makes direct comparison challenging. However, the meta-analysis highlight the potential efficacy of other, more actively involved telemedicine interventions to increase CPAP adherence [20].

In relation to the present study, it is relevant to contemplate the role of telephone consultations in fostering increased adherence. Notably, our study did not incorporate ‘active’ interventions aimed at influencing participants towards enhanced adherence, such as automated messages or outpatient clinic phone calls for nonadherent individuals. The primary outcome revealed no significant difference in adherence to CPAP therapy between the TC and SC group. However, a higher proportion of participants in the TC group demonstrated compliance with consultations compared to the SC group. It is likely that non-adherent participants in the SC group may have been more inclined to withdraw from the study to avoid follow-up consultations. In contrast, the TC group encountered a considerably less intrusive action on their everyday life through telephone calls. This circumstance likely contributed to the higher completion rate in the TC group although resulting in a diminished utilization of CPAP devices. This observation is supported by a study [22], which indicated that participants viewed video consultations favorably as a substitute for face-to-face consultations, due to convenience and time efficiency. Nevertheless, as highlighted in the previously mentioned study [22], it is important to note that some participants express a preference for face-to-face consultations, and others encounter technical difficulties with video consultations. These factors could potentially influence our study outcomes, particularly considering that the SC group had a median of fewer additional consultations than the TC group, though not statistically significant. This underpins that telemedicine cannot completely replace face-to-face consultations [22], emphasizing the dependency on individual patient preferences and treatment-related issues.

The varying results across our study and others using telemedicine interventions may originate from various determinants influencing adherence and non-adherence. Notably, participants who completed the study exhibited a significantly higher baseline AHI compared to those who dropped out, suggesting that a high AHI may increase the likelihood of patient adherence to treatment. A sub-analysis was conducted to examine adherence among patients categorized into mild, moderate, and severe obstructive sleep apnea (OSA) groups. It was observed that adherence rates measured in percentage improved progressively from the mild to the moderate and then to the severe OSA groups, in the unadjusted analysis. However, this trend reached statistical significance (*p* = 0.016) only when considering the entire cohort of participants. When the analysis was confined to either TC or SC groups individually, significance was not achieved, possibly due to the smaller size of these subgroups. This finding aligns with a Danish longitudinal study [11], which identified a positive association between severe AHI and increased adherence to CPAP use. While the literature recognizes the impact of perceived effectiveness and treatment side effects on adherence [9], our study did not specifically address these determinants in the interventions comparing adherence between TC and SC. Our findings showed that using TC as a substitute for SC might retain patients in the out-clinics. This observation is supported by Woehrle et al. 2017 who found that a telemedicine-based proactive strategy using the Airview™ device for the management of patients receiving PAP therapy, had a decreasing effect on the therapy termination rate over time, regardless of gender, device used and insurance type [23]. Having the ability to retain patients in treatment, might after all provide a foundation for offering patients other interventions that contribute to increased adherence to CPAP therapy.

### Strengths and limitations

Strengths of this study include its randomized controlled trial design, offering robust evidence for evaluating intervention effectiveness compared to a control group. Also, using intention-to-treat analysis for the primary outcome aligns with CONSORT recommendations [24], enhancing internal validity. Additionally, the blinding of data assessor minimizes detection bias, contributing to the overall reliability of the study [25]. However, the study is not without limitations. Despite randomization, there is a risk of selection bias due to the potential ability of clinical staff to predict the last assignments of each block randomization. The intention-to-treat analysis was challenged with missing data regarding CPAP use from dropouts and loss to follow-ups. Therefore, a worse-case scenario was conducted assessing dropouts as non-adherents. However, this might have led to type 2 errors in the primary outcome´s effect estimate, as it is likely that some of the dropouts continued treatment even though they did not continue in the study. The substantial dropout rate resulted in baseline differences in the population of the complete case analysis, potentially limiting the generalizability of findings to specific subgroups. Despite regression analyses adjusting for covariates, the risk of residual confounding influencing the results persists. While the study population characteristics align with other Danish longitudinal studies [11, 26], caution is needed in interpreting the findings due to limitations in effect estimates.

## Conclusion

The study found that TC did not affect adherence to CPAP therapy compared to SC significantly, and thereby might be considered as an alternative to in-person consultations to some extent. We found a higher compliance rate for follow-up consultations among participants in the TC group. This suggest that substituting TC for SC might retain patients in the outpatient clinics, which could serve as a basis for providing additional interventions to patients that support better adherence to CPAP therapy. Furthermore, the only influencing factor found was a higher baseline AHI among participants who completed the study compared to dropouts, indicating that a high AHI may increase the likelihood of patient adherence to therapy.

### Implications for practice and research

If telephone follow-up become a part of CPAP therapy management, it is important to carefully consider patient characteristics and treatment-related issues to decrease potential decline in adherence. New studies should explore alternative interventions that might increase adherence to CPAP therapy, and additionally identify patient characteristics related to nonadherence to CPAP therapy and noncompliance with follow-up consultations.

### Supplementary Information

Below is the link to the electronic supplementary material.

Supplementary file1 (DOCX 15 kb)

## Data Availability

The datasets generated and analyzed during the current study are available from the corresponding author on reasonable request.

## Abbreviations

OSA: Obstructive sleep apnea
CPAP: Continuous positive airway pressure
AHI: Apnea-hypopnea-index
ESS: Epworth sleepiness scale
SC: Standard out-clinic follow-up consultation
TC: Telephone follow-up consultation
