# Supplementary material for: Telephone follow-up as a substitute for standard out-clinic follow-up in CPAP therapy for obstructive sleep apnea patients: a randomized controlled trial
Source: Sleep Breath. 2024 May 8;28(4):1651–9. doi: 10.1007/s11325-024-03045-w (PMC11303568; doi:10.1007/s11325-024-03045-w)
Supplement: Supplementary file 1 — Supplementary file1 (DOCX 15 kb) [file 11325_2024_3045_MOESM1_ESM.docx]

**Supplementary document 1** Dropout analysis presenting baseline demographics and sleep data for participants who completed the study and dropped out during the study

|  | **Completed study** | **Dropped out** | **P-value** |
| --- | --- | --- | --- |
| **n (%)** | 140 (70.0) | 60 (30.0) |  |
| **Groups** |  |  | 0.064 |
| SC group | 64 (32) | 36 (18) |  |
| TC group | 76 (38) | 24 (12) |  |
| **Sex** |  |  | 0.152 |
| Male | 107 (76.4) | 40 (66.7) |  |
| Female | 33 (23.6) | 20 (33.3) |  |
| **Age** | 56.2 (12.1) | 53.1 (12.6) | 0.103 |
| **BMI** | 31.92 (5.45) | 31.03 (5.17) | 0.287 |
| **Distance to hospital (km)** | 27 (18-37) | 27 (15-34) | 0.515 |
| Missing | 1 (0.7) | 1 (1.67) |  |
| **Baseline AHI** | 33 (21-49.5) | 18.5 (13.5-33.5) | <0.001 |
| **Baseline ESS** | 9 (5-12) | 8 (4-12) | 0.45 |
| Missing | 1 (0.5) | 0 (0) |  |

Categorical variables are n (%) and continuous variable are mean (SD) for parametric data and median (25th-75th percentiles) for non-parametric data.

Abbreviation: SC (standard out-clinic follow-up consultation); TC (telephone follow-up consultation); OSA (obstructive sleep apnea); AHI (apnea-hypopnea-index); ESS (Epworth sleepiness scale).
